# Tipping points induced by palaeo-human impacts can explain presence of savannah in Malagasy and global systems where forest is expected

**DOI:** 10.1098/rspb.2021.2771

**Published:** 2022-03-30

**Authors:** Grant S. Joseph, Andrinajoro R. Rakotoarivelo, Colleen L. Seymour

**Affiliations:** ^1^ FitzPatrick Institute of African Ornithology, DSI-NRF Centre of Excellence, Department of Biological Sciences, University of Cape Town, Rondebosch 7701, South Africa; ^2^ Afro-mountain Research Unit, The University of the Free State Qwaqwa, Private Bag X13, Phuthaditjhaba 9866, Republic of South Africa; ^3^ Natiora Ahy, Lot IIU57 K Bis, Ampahibe, Antananarivo 101, Madagascar; ^4^ South African National Biodiversity Institute, Kirstenbosch Research Centre, Private Bag X7, Claremont 7735, South Africa

**Keywords:** alternate stable states, annual rainfall, C_4_-grasses, fire, Madagascar, savannah and forest boundary

## Abstract

Models aimed at understanding C_4_-savannah distribution for Australia, Africa and South America support transition to forest at high mean annual precipitation (MAP), and savannah grasslands of Madagascar have recently been reported to be similarly limited. Yet, when savannah/grassland presence data are plotted against MAP for the various ecosystems across the Malagasy Central Highlands, the relationship does not hold. Furthermore, it does not always hold in other sites on other continents. Instead, in high-rainfall savannahs, palaeo-human impacts appear to have selected a fire-adapted habitat, creating tipping points that allow savannah persistence despite high rainfall, suppressing forest return. We conducted the largest systematic literature review to date for global evidence of palaeo-human impacts in savannahs, and conclude that impacts are widespread and should be incorporated into models aimed at understanding savannah persistence at elevated precipitation, particularly as more palaeodata emerges. Building on existing studies, we refine the MAP savannah relationship at higher MAP. Palaeoanthropogenic impact can help explain inconsistencies in the savannah/forest boundary at higher MAP, and points to a key role for palaeoecology in understanding systems. Including these effects presents a crucial change to our understanding of factors determining global savannah distribution, supporting a human hand in much of their formation.

## 1. Background

The transition between savannah grassland and forest is influenced by mean annual precipitation (MAP): rainfall below approximately 650 mm yr^−1^ restricts woody cover; higher rainfall facilitates canopy-closure and forest formation, when intensive fires and herbivory are absent [1]. A decade ago, a framework (henceforth referred to as ‘the framework’) was developed that addressed the cyclical and reversible flux of savannah grassland and forest as alternate stable states [2]. According to this model, annual precipitation, and to a lesser degree, seasonality, soil fertility and continent (and therefore evolutionary history) determined the probability of natural savannah occurrence and fire frequency [2]. The framework has dominated the discourse surrounding global savannah distribution [2–7], with increasing MAP emerging as a central factor ‘driving the transition from savannah to forest on the mesic end of the continuum’ [2, p. 200]. The framework posits that the probability of closed-canopy forest approaches 100% at 1750 mm for Africa, at 2000 mm for Australia and 2500 mm for South America, with the probability of savannah grassland occurrence being negligible beyond these higher MAP levels. Recently, it was reported that ‘modern Malagasy grassy ecosystem limits are like Australia … Africa and South America’ [3, p. 2] and are limited in a similar fashion, but with grassland persisting at higher MAP (probability of grassland occurrence exceeded 15% even at MAP of approximately 3000 mm [3], suggesting that Madagascar harbours the wettest mesic savannah globally). Although mainland Africa is only 500 km distant, African savannah grasslands transition to forest at half that MAP [2].

Such high probabilities of savannah occurrence at MAP three to four times greater than that which can theoretically support forest [1] is intriguing, given recent findings from the 200 000 km^2^ Malagasy Central Highlands (MCH), Madagascar's largest phytogeographical biome. Encompassing almost half the island and containing a multitude of ecosystems, including grasslands, forests, woodlands and ericoid habitats, the data show that the probability of savannah being limited at high MAP is negligible, and the transition to forest as plotted for Africa, Australia and South America [2] does not occur [6]. Indeed, there is little transition to any other habitat type; less than 2% of the system is forested [8], and the remainder is dominated by crops and treeless grassland (despite MAP ranging from 764 mm to greater than 3200 mm [9]). If MCH grasslands were limited at the mesic end of the spectrum by high MAP in similar fashion to other mesic systems and adapting the same probabilities as plotted island-wide, almost half the MCH would be a closed-canopy system, and not treeless grassland [3,8].

The failure of transition at high MAP has been observed elsewhere, and a decade ago, the role of palaeo-human activity in explaining these patterns was considered, although the idea has not received widespread attention. Lloyd *et al.* [10] questioned (i) whether persistence of almost treeless savannah grasslands at extremely high MAP that should support forest (e.g. those contiguous to Africa's Congo rainforests, and South American Sipaliwini, Rupinuni and Rio Branco savannahs, where MAP can exceed 3000 mm) might be due to historic anthropogenic activity, and (ii) whether this phenomenon is widespread. At the time of publication (2008), conclusive palaeoecological evidence for frequent human-lit fires that permanently decouple the savannah-forest alternate state from MAP had yet to emerge. Subsequently, evidence for historical human transformation of forest to savannah has mounted [11–18], and new lines of multidisciplinary data have emerged, spanning archaeological, geochronological, geomorphological and palaeoenvironmental disciplines [19,20]. These data call into question the reversibility of a predictable cycle of forest dominance in wet periods, with a grass-rich, open-canopy or more sclerophyllous habitat in drier times, in situations where there has been large-scale and protracted conversion of forest to open habitat following human settlement [12–14,16,19,21].

For example, recent findings from African palaeocores support repeated human-lit fires inducing a ‘permanent tipping point’, whereafter irrevocable habitat transformation decouples the savannah-forest cycle from MAP [19,20]. Forest recolonization is forever prevented, so that subsequent periods of high MAP produce only alluvial fans (signifying erosion) and persistent savannah [19]. The suite of fire-adapted grasses and shrubs that emerges is less diverse and different in structure from any previous habitat [19,20]. Shifting the focus to Madagascar, reliable MAP records and palaeo-data on the MCH provide an ideal opportunity to test for palaeoanthropogenic decoupling of the savannah-forest alternate stable state to explain savannah persistence at MAP where forest would be highly probable in Australian, African and South American mesic systems [2]. Although human arrival times remain contentious for Madagascar as a whole (potentially dating to around 10 ka [22]), there is good palaeoecological and archaeological evidence for later settlement of the MCH 1.5–2 ka [23]. For the MCH, there is (i) a palaeocore dating to 154 ka with habitat patterns that closely parallel aforementioned cores from mainland Africa [19,20], with drier periods being more grassy and wetter periods more forested, until human arrival [21]; (ii) well-documented human presence over the last 1.5 ka [23]; (iii) charcoal deposits coeval with human settlement at MCH palaeocore sites marking conversion of forest, woodland and ericoids [21,24] to what is now a treeless grassland around 1.5 ka; (iv) selection of dominant grass species by human-lit fire and spread of dominant grass species with pastoralism [6,7] and (v) persistence of treeless savannahs at exceptionally high MAP following human settlement [6,25]. Notably, this system is novel: grassland largely devoid of associated arboreal and/or ericoid pollen is without parallel in the palaeocore, as previous, presettlement grassy periods tended to characterize drier periods, and harboured a diversity of arboreal and/or ericoid pollen [21,26].

Lehmann *et al.* [2] in 2011 noted, ‘if savannas in different parts of the world have different environmental limits, we need to search for alternative ecological explanations or turn to historical differences to explain these divergences' (p. 205). Without doubt, informed and appropriate management and conservation needs better understanding of vegetation–disturbance interactions and feedbacks between woody plants and grasses [3]. Here, we aim to contribute to understanding these systems, and address occurrence of savannah where the framework predicts forest [2,3]. We build on the existing framework by introducing a new variable: palaeoanthropogenic impact of the type that irrevocably severs the relationship between MAP and the likelihood of savannah or forest presence [19,20]. We interrogate the issue across mesic savannahs, as understanding factors that delimit habitat is of global conservation importance. Given all mesic savannahs have been subjected to some degree of historic and intensive human impacts, identifying whether these impacts disrupt alternate stable states would reshape our concept of limitations to alternate stable states involving distribution of savannah grassland, savannah woodland and forest systems, globally. Here, we:

(i) compare MAP limitation to the probability of savannah grassland occurrence across the ecosystems spanning the MCH, with those recently published for Madagascar [3]. In natural savannah systems, the 2011 framework supports an increased probability of closed-canopy forest at high MAP where the forest-savannah alternate stable state cycle holds [2]. Absence of limitation would support crossing of a threshold, beyond which MAP no longer influences likelihood of savannah or forest persistence with any accuracy [20];
(ii) collate MAP for extant MCH forest patches, anticipating a low representation of savannah with MAP approaching 3000 mm, and if MAP limits savannah grassland in similar fashion to Australia, Africa and South America, as reported [3], an increased probability of forest at higher MAP;
(iii) investigate whether high MAP limits fire frequency on the MCH, given the current framework predicts that MAP between 1750 and 2500 mm limits fire across mesic savannah systems globally [2]. Decoupling of fire from MAP in the context of palaeoanthropgenic evidence would lend support to ancient human impact as a variable in future modelling of the distribution and persistence of savannahs at MAPs that should support forest; and
(iv) assess for global evidence of decoupling of savannahs from MAP where palaeocore data exist, and whether human activity in the past may have driven these changes, by conducting the largest systematic literature search on this subject to date, and combining this with satellite data.

## 2. Material and methods

### (a) Probability of savannah and forest relative to MAP across the MCH

We follow Lehmann *et al*. [2] in evaluating how the mesic aspect of the MAP spectrum impacts savannah, which transitions with increasing probability to forest at high rainfall in Africa, South America and Australia. We emphasize this to distinguish it from events at lower MAP, where savannah grassland transitions to ‘arid shrubland/thicket/spinifex on the arid end of the continuum’ [2, p. 200]. Changes at lower rainfall are not tested here (pertaining to MAP ranges beyond the scope of this study). Across the MCH, MAP follows gradients from west (less than 1600 mm) to east (greater than 2500 mm) and south (less than 1200 mm) to north (greater than 2500 mm), with minima and maxima of 764 mm to greater than 3000 mm [4,9,27]. Today 80% of the MCH is treeless grassland, dominated by cosmopolitan, fire-adapted and/or ungulate grazing-adapted grass species (e.g. *Loudetia simplex*, *Trachypogon spicatus* and *Schizachyrium sanguineum*) [4,6,7,28]. Vast obligate grazing lawns [5] probably represent anthropogenic formations selected through pastoralism over the past millennium, as the dominant grasses have traits selected by broad-muzzled obligate grazing ungulates [5,6]. Evaluation of all MCH subfossils found to date confirms no endemic specimen was an obligate C_4_-grazer, nor even a predominant C_4_-feeder, of the type that selects obligate grazing lawns [6,29]. Most species were C_3_-woody feeders, and the mixed-feeders with the greatest C_4_ dietry content, elephant birds [29], were bill-feeders that would probably have selected for different traits [6]. Woody habitat accounts for less than 5% of MCH habitat and occurs in two principal forms: closed-canopy evergreen forest, and tapia, an open-canopied woodland [4,8], described as ‘an anthropogenic formation, modified from presettlement forests by burning and cutting’ [30, p. 327].

**Figure 1.**
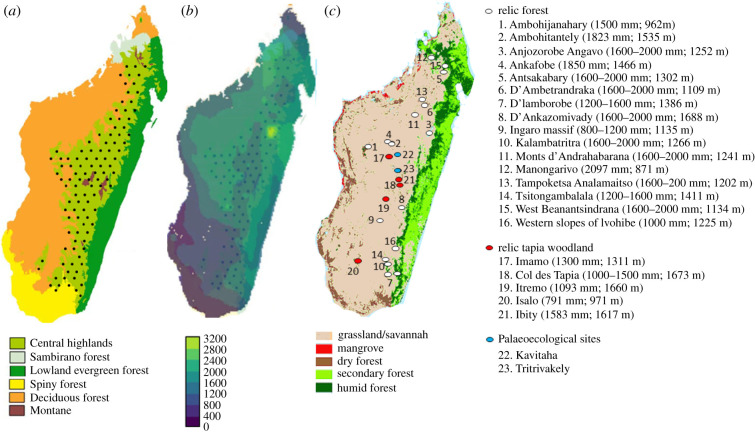
(*a*) Schematic grid overlayed on the Malagasy Central Highland (MCH) phytogeographic region. (*b*) Mean annual precipitation (MAP, in mm) superimposed on the grid (modified from [9,33]). (*c*) Remotely sensed habitat (modified from [31,33]); MAP and altitude (mm; m) are listed for MCH forest and woodland sites, corroborating grassland-limitation at far lower MAP than reported for a pan-Malagasy dataset [3]. (Online version in colour.)

To sample random points across the MCH to assess savannah/forest habitat relative to MAP, we superimposed a grid of 106 focal points, each comprising four geographical coordinates spaced *ca* 40 km apart, over a remotely sensed vegetation map [31,32] (figure 1). We followed Lehmann *et al*. [2], classifying savannah as a dominant C_4-_grass layer with discontinuous tree cover. Vegetation at each point was recorded (closed-canopy forest or savannah grassland). Coordinates coinciding with riparian cover, plantations of alien trees, elevations greater than 1800 m (signifying the distinct montane biome), shrubland, thicket, cropland and heath (which do not represent an exposed C_4_-layer) were excluded, and another random point was taken, where possible. ‘Closed-canopy’ comprised either ‘forest, dry forest, rainforest, evergreen tropical or moist tropical forest and closed forest’ [2]. As the MCH falls between 800 and 1800 m, we followed the Worldwide Fund for Nature by incorporating areas in central Madagascar classified as subhumid forest, providing they fell within these altitudinal parameters (and so included forests at relevant altitude that represent transition between the Sambirano and MCH phytogeographic zones, e.g. Manongarivo Forest [34]). If no available natural/non-agricultural/non-residential habitat was present beyond a 20 km radius from the focal point, that geographical coordinate was omitted. Much of the MCH is cropland, so 99 of the 424 geographical coordinates were unsuitable. As little of the MCH closed-canopy forest remains, and as our screening survey [6] was almost entirely composed of savannah sites, we ensured the representation of closed-canopy cover by incorporating at least one geographical coordinate from each of the 16 recognized forests listed in figure 1. This approach potentially creates a biased impression of greater forest cover in the MCH, but as the 2021 Malagasy dataset supports a probability of savannah occurrence with increasing MAP of roughly 50% at MAP of 1750 mm, 30%, at 2000 mm and 15%, above 2500 mm [3], and as the study reported that Malagasy systems are limited in a similar fashion to other mesic savannahs (which at the mesic aspect of the MAP spectrum transition to forest [2]), we included forest patches where present, anticipating a 50% probability of forest at MAP of 1750 mm, 70%, at 2000 mm and 85%, above 2500 mm. Using these criteria, we generated 325 coordinates across the MCH. We then superimposed MAP [9] to the grid (figure 1), and the 325 coordinate dataset was binned into MAP ranges at 400 mm increments (i.e. 0, 400, 800, 1200, 1600, 2000, 2400, 2800 and 3200 mm [33]). Within each bin, probability of savannah presence was calculated following Lehmann *et al.* [2], where the mean of all points within that rainfall bin reflects the probability of presence of savannah (0, no savannah; 1, savannah; figure 1; electronic supplementary material, S1).

### (b) MAP where forest occurs on the MCH

To evaluate for increased probability of MCH forest at higher MAP, we tabulated MAP for remaining patches of forest on the MCH.

### (c) Probability of fires relative to MAP across the MCH

The existing framework supports limitation to fire where MAP exceeds 2000–2500 mm across savannahs globally [2], yet fires are frequently observed in Madagascar where MAP exceeds 3000 mm (G.S.J. *et al.* 2019, personal observation). We evaluated MAP-related limits to fire on the MCH, using the same 325 geographical coordinates described above. We plotted probability of fire relative to MAP by superimposing Moderate Resolution Imaging Spectroradiometer (MODIS; https://firms.modaps.eosdis.nasa.gov/) fire data across MAP gradients [35]. Only fires occurring within 1 km^2^ of the central pixel were included, and MODIS fires with confidence lower than 50% were excluded. Thus the dataset probably underrepresents fire occurrence (electronic supplementary material, S2 and S3). Following Lehmann *et al.* [2], if a coordinate burned within a 9 yr period (ending in 2020), we classified fire occurrence as one (and zero if not). Probability of fire was the mean of points within that rainfall bin [2].

### (d) High-rainfall savannahs and palaeocores supporting irrevocable habitat transformation: the global scale

We conducted a systematic literature review for relevant palaeorecords from high-rainfall savannahs where the framework plots high probability of limitation of savannah, and transition to forest (i.e. MAP exceeding 1750 mm for Africa, 2000 mm for Australia, 2500 mm for South America and 2750 mm for Madagascar [2,3]), using the snowball method [6,36]. We searched Google Scholar (from June to September 2021), using the search terms: ‘savannah* OR grassland* AND paleoecology* OR palaeoecolog* AND human* OR anthropogen AND disturbance* or fire* AND origin*’. The primary search, to a minimum of 40 references, was stopped when additional searching exceeded 75% of studies already included as pertinent but provided no additional applicable references. We then repeated the method, limiting the search to 2020 and 2021, to maximize capture of less-cited recent studies, which can be assigned lower priority by search engines (electronic supplementary material, S4). References cited in relevant studies from the primary search were then examined to detect additional studies. Although the literature search cannot ensure all relevant studies were included, systematic use of terms ensured a replicable, representative set of studies [6,36,37]. We then evaluated these for evidence of decoupling of savannah from MAP in the savannah systems identified from the literature search, plotting visually determined percentage-savannah cover (using magnified satellite imagery verified with habitat maps) against MAP gradients for the systems identified [11–14,19,20,32,38].

## 3. Results and discussion

### (a) Probability of savannah and forest relative to MAP across the MCH

**Figure 2.**
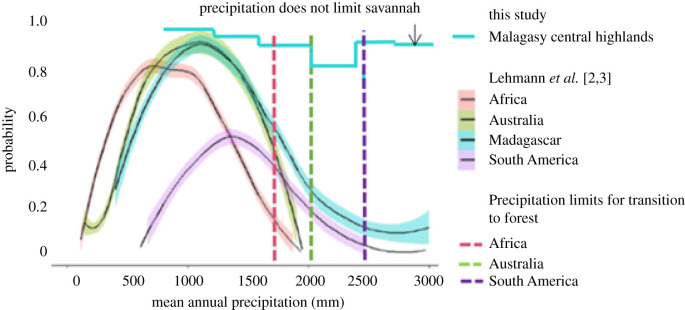
Probability of savannah presence as a function of mean annual precipitation (MAP). Unlike Africa, Australia and South America [2], the probability of savannah for Malagasy Central Highlands (MCH) was decoupled from limiting effects of high MAP. Instead of the 15% probability of savannah at MAP beyond 2500 mm reported for all of Madagascar [3], probability exceeded 80% on MCH. Forest, predicted by the framework at high MAP in other mesic systems [2], was largely absent. (Online version in colour.)

Although probabilities of savannah limitation for Madagascar were reported at roughly 50% when MAP reached 1750 mm, 70% for 2000 mm, and 85% for beyond 2500 mm [3], this did not hold for the MCH (figure 2). In African, South American and Australian systems, the transition at this mesic aspect of the MAP spectrum is to forest [2], yet despite reports that Malagasy savannah grasslands are similarly limited by MAP [3], there was no evidence of transformation to forest or to any other natural habitat. Instead, habitat on the MCH was decoupled from MAP. Forest occurrence was less than 5% across the entire MAP range, and at MAP levels where presence of grassland was plotted with a 15% probability, the opposite was found on the MCH: grassland presence exceeded 80% of cover, when African, South American and Australian systems have an 85% probability of forest being present.

At this juncture, we emphasize a growing need for clear definition of ecological terminology. In the original 2011 framework [2], the term ‘savannah’ is used, whereas in the 2021 iteration, ‘grassland’ is preferred [3]. Although some authors use these terms interchangeably [2,3], we propose the use of (i) ‘savannah’ as an umbrella term to describe a system characterized by a C_4_-grassy layer, with varying degrees of woody cover, vacillating with rainfall, fire, herbivory and soils, ranging from almost treeless grasslands (at low MAP and frequent fire), to woodlands with canopies that are close to closure (at higher MAP and little fire) [1]; (ii) ‘savannah woodland’ to describe only savannah dominated by the woody component; and (iii) ‘savannah grassland’ to describe only savannah dominated by grasses, that is nearly treeless [1–6,10,26,28]. This is important, as these alternate stable states support not only different patterns and processes and unique biota [39], but their functional ecology also differs [5,40]. Furthermore, we recommend C_3_-dominated grasslands be classified as such (C_3_-grasslands), to avert confusion.

### (b) MAP where forest occurs on the MCH

Grasslands transition on the MCH at far lower MAP than suggested by the recent study, and forest arises between 800 and 2097 mm, with the majority of relic patches occurring between 1600 and 2000 mm, similar to the transition point to forest (1750 mm) for African savannahs [2] (figure 1). Forest persistence at low MAP (approx. 800 mm) corroborates Sankaran *et al*. [1]. Indeed, as grassland is limited at far lower MAP than the anticipated 3000 mm [3], mainland Africa offers a better fit for the MCH [2]. MAP across most of the MCH is 1200–2500 mm [9,27], so application of the African curve (which fits rainfall data for relic MCH forests) to the MCH supports even higher probabilities of forest: 60% at 1300 mm, 80% at 1400 mm and greater than 95% at 1750 mm. Under this scenario, most of the MCH should be forest, not treeless savannah, at current MAP. It is noteworthy that at elevations approaching and exceeding 1700 m, there is also support for greater MCH ericoid habitat than seen today [6].

In Africa (Malawi), similarly surprising decoupling of habitat from rainfall has been observed. Frequent palaeoanthropogenic fires have been shown to establish a tipping point, beyond which the cycle of forest recolonization becomes permanently broken. First, a crash in podocarps occurred [19]. Next, a biotically depauperate system dominanted by fire-adapted grasses, olive trees, and Asteraceae emerged [20], following which a return to high MAP no longer promoted forest or woodland, but rather alluvial fans, signifying erosion, and persistent fire-adapted grassland [19,20].

**Figure 3.**
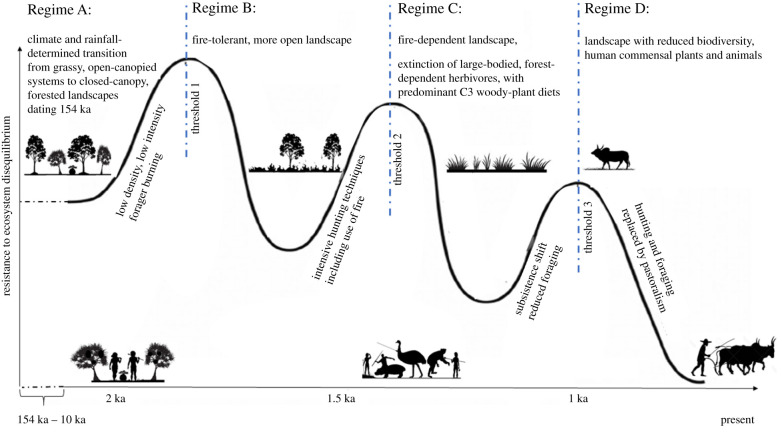
Conceptual model of changes in ecosystem state in the Malagasy Central Highland (MCH), similar to those outlined for mainland Africa by Thompson *et al*. [19,20]. Ecosystems cross cumulative thresholds with time, resulting in a tipping point, whereafter vegetation differs from any preceding habitat [19,20]. The MCH palaeocore reveals a cycle of climate-driven vacillation of forest, ericoids and grassy components (Regime A), that lasts for 154 000 years [21]. This becomes disrupted following human settlement (Regime B, C, D). The current grassland is largely treeless, depleted of ericoid and/or forest pollen that had co-occurred in previous grassy periods; it does not revert to forest even at levels of annual rainfall that provide a 90% probability of transition of African, Australian and South American systems to forest. This shift to grass pollen permanently replacing tree and ericoid pollen appears to have been island-wide, around 1 ka [24,43–45]. (Online version in colour.)

The MCH shows remarkable parallels. Pre-Holocene vegetation shifted with climate, from forest to woodland and ericoids, and the relative percentage of grass pollen increased in drier periods [21]. When human occupation intensified around 1.5 ka [23], charcoal deposits at Kavitaha and Tritrivakely support coeval burning of forest, whereafter forest and ericoid pollen collapsed from 80% to only negligible proportions of total pollen, and grass pollen rose from 15% to greater than 80% [21], today approaching 100% across a treeless grassland. The process mirrors African cores [19,20]. Similar genera and families of forest trees, most notably podocarps [21], have been lost, and fire-adapted grasses, Asteraceae, and local olive trees persist [21,41]. On MCH, erosion gullies called *lavaka* (literally ‘holes’, up to 30 km^2^ in area), are prominent, and although the process is likely multifactorial, overly frequent fire corresponding to humans shifting from hunting to farming likely contributed to their formation and spread [42]. The emergent system has no precedent in the core dating to 154 ka (figure 3). Previous grassy periods were characterized by some degree of ericoid or arboreal pollen, and diversity of tree pollen was rich [21].

The remaining MCH relic forests possess 25 m canopies harbouring epiphytes, dense understories and at elevation, mosses and lichens [27] and are characterized by fire-sensitive tree species that lack thick-bark, resprout poorly post-fire and do not exhibit clonal spread [46]. Many co-occur in eastern lowland or Sambirano rainforests and riparian areas (with occasional pockets along remote streams and in slope hollows), such as *Podocarpus madagascariensis* (Podocarpaceae), *Ficus botryoides* (Moraceae), *Gambeya boiviniana* (Sapotaceae), *Dalbergia madagascariensis* (Leguminosae), *Baronia taratana* (Anacardiaceae), *Tambourissa hildebrandtii* (Monimiaceae), *Uapaca densifolia* (Phyllanthaceae), *Nuxia capitata* (Stilbaceae), *Homalium nudiflorum* (Salicaceae), *Leptolaena pauciflora* (Sarcolaenaceae), *Neocussonia longipedicellata* (Araliaceae) and *Canarium madagascariense* (Burseraceae) [6,46] (electronic supplementary material, S5).

Tapia, a sclerophyllous woodland selected by human-lit fires for farming silkworms and for pasture comprises less than 2% of the MCH and is dominated by thick-barked, fire-resilient *Uapaca bojeri* with a grassy understory; Sarcolaenaceae and other trees co-occur where less frequent fires prevail [6,30,47]. Indeed, the principal threat to *Schizolaena microphylla* and *Xerochlamys diospyroidea* is fire [46]. Savannah and drought-adapted Oleaceae adjust to natural fire and grazing through clonal growth (e.g. stump area reaches 80 m^2^ in *Olea europaea* subsp. *laperrinei*), so clonal growth was anticipated for *Noronhia lowryi*, the only species of 83 Malagasy Oleaceae to occur in tapia stands (most are forest-adapted) [41].Yet stump area never exceeded 1 m^2^, a response more in line with expectations for genetically affiliated forest olives like *N. brevituba*. It follows that *N. lowryi* is ‘endangered due to frequent human-induced fires’ [41, p. 229], and limited fire-adaptation is supported by a population bottleneck that may have coincided with fires following human arrival [41].

Although considered by some to be natural savannah trees [4], might *U. bojeri* represent anthropogenically selected survivors of more closed-canopy formations? Other *Uapaca* species occur in species-diverse, complex woodlands and forests in both Africa and Madagascar. Thick, fire-resistant bark is shared with *U. kirkiana*, which occurs naturally in African lowland forests (and woodland), where forest-burning can preferentially select it [6,47]. Anthropogenic fires have removed historic MCH tree structure [6,30,48]; palaeo-pollens affirm forest-associated tree families occurred where today there is treeless grassland (e.g. Aquifoliaceae, Malvaceae, Meliaceae, Moraceae Podocarpaceae, Phyllanthaceae, Sapindaceae and Sapotaceae [21]). It is notable that many species (e.g. *Sarcolaena oblongifolia*, *Leptolaena pauciflora, Aphloia theiformis* (Aphloiaceae) and *Baronia taratana* (Anacardiaceae)) that are absent from frequently burned tapia stands co-occur in fire-sensitive gallery forest [46]. Furthermore, the less modified tapia-like ‘western slopes sclerophyllous forests' sustain 18 tree families and 26 genera [30], emphasizing greater historic complexity. Co-occurrence of many woodland species in gallery rainforest and limited fire-adaptation relative to *U. bojeri* support a human-induced shift from fire-sensitive species to increasingly fire-adapted assemblages, culminating in vast fire-adapted, treeless grasslands. The disappearance of fire-sensitive forest species, and their failure to return despite current rainfall supporting forest, underscores establishment of a tipping point (figure 3), and frequent fires now threaten even the remaining *U. bojeri*, decreasing seed set and recruitment, and increasing mortality [47].

Island restriction and intensity of disturbance may have contributed to the rapid change. New Zealand, although a very different system to Madagascar, provides an analogy. Mostly forested when humans arrived 800 years ago, it is now anthropogenic grassland dominated by a historically insignificant native grass genus (*Chionochloa*), selected by anthropogenic deforestation, human-lit fire and introduced grazers [40,49].

### (c) Probability of fire relative to MAP across the MCH

**Figure 4.**
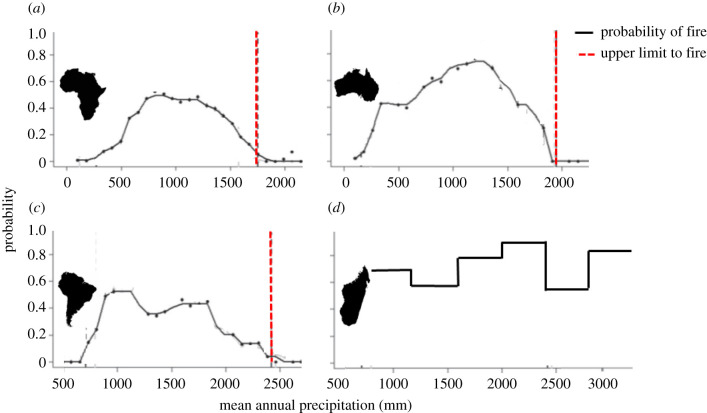
Probability of fire as a function of mean annual precipitation (MAP). The existing framework [2] supports MAP limiting probability of fire in (*a*) African, (*b*) Australian and (*c*) South American savannahs to less than 5% by 2500 mm (broken red lines). Yet in (*d*) Malagasy Central Highlands, the probability approaches 80% even at MAP exceeding 3000 mm, suggesting decoupling of the relationship between fire and MAP. (Online version in colour.)

Although the current framework supports less than 5% probability of fires across mesic savannahs when MAP reaches 1750–2500 mm [2], on the MCH, fire is decoupled from MAP. Indeed, likelihood of fire approaches 80% even where MAP exceeds 3000 mm (figure 4). This is remarkable, as high rainfall should reduce the likelihood of burning [2]. This probably reflects both historic and current events, because fires are lit annually to bi-annually for grazing, and the practice dates to early settlement, accounting for 95–99% of all fires in terms of surface area burned [35,50]. The MCH burns despite receiving far higher MAP than that of nearby African savannah grasslands, because only 1–5% of fires are natural [50].

Remotely sensed daily fire-events from 2012 to 2020 produced a 550 000-event dataset for Madagascar [35] over 9 years. Fire is central to anthropogenic transformation of forest to savannah, and the process can be rapid. Lowland Malagasy forest converts in only 30 years of cutting and burning across seven fallow cycles, to a species-impoverished successional C_4_-grassland, similar to that found on MCH, also characterized by erosion gullies [48]. The MCH has been subjected to human burning for greater than 1.5 ka [24,50]. MCH grasslands are dominated by a handful of cosmopolitan grass species, and the most dominant, *Loudetia simplex*, has likely spread recently (1 ka) and widely (across the northern MCH) with pastoralism and human-lit fire [7]. This supports Bond *et al*. [51, p. 1746], who noted: ‘If grasslands originated, or had expanded from small areas only in the last two thousand years, we would also expect there to be few species and genera with very wide distribution.’ Our results emphasize a need to re-evaluate not only savannah persistence at high rainfall, but also fire decoupling from limiting effects of high MAP, as this too may apply across other ecosystems.

### (d) High-rainfall savannahs and palaeocores support irrevocable habitat transformation at a global scale

**Figure 5.**
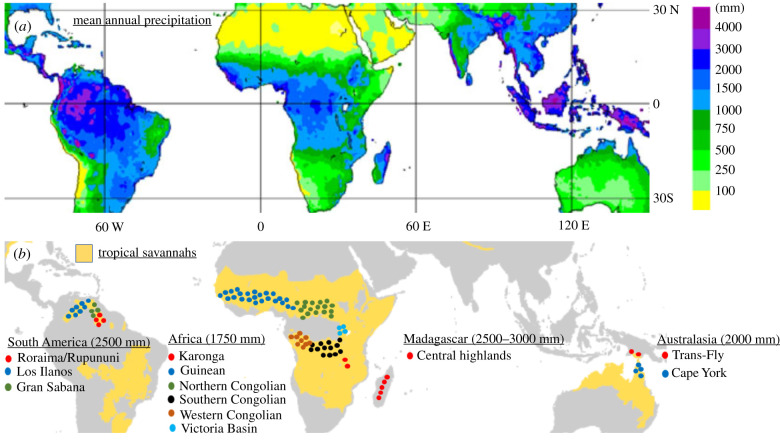
(*a*) Mean annual precipitation (MAP), GPCC V2011 [52]. (*b*) Savannah systems [2,53] containing broad areas in which MAP (in brackets) levels support a high probability of forest [2]. The Malagasy Central Highlands is not shaded, reflecting the habitat dispute (Olson *et al*. [53] 2001 chart broad-leafed forest, Lehmann *et al*. [2] 2011 map savannah and Joseph *et al*. [6] 2021 plot a complex mosaic comprising expansive forests at higher MAP, ericoid habitat at altitude, and savannah woodland at lower MAP). (Online version in colour.)

Of 1931 references inspected, 81 relevant studies were identified, representing the largest review on the subject to date. In all, 184 references from the 81 studies mentioned palaeoanthropogenic influences across modern savannahs where forest would be expected. Of the 184, 64 represented South American systems (59 for the Gran Sabana of Venezuela, 3 for the Cerrado and 2 for Columbian savannahs); 37 represented Madagascar (all on the MCH); 20 represented African (spanning peri-Congo, Guinean and Malawi savannahs); 11 Australian and 2 Papua New Guinean savannahs. An additional 50 references applied globally. Twelve systems (covering an area exceeding 2 million km^2^; electronic supplementary material, S1) harboured savannahs at levels predicted by the framework to be forest (figure 5). However, only three areas could be reliably identified as having strong and compelling evidence for (i) irrevocable change induced; (ii) following well-documented palaeo-human impacts to vegetation; (iii) with rainfall ranges; (iv) coinciding with the upper MAP levels delineated by Lehmann *et al.* [2]. This points to a need for additional palaeocore data to investigate this phenomenon at other sites across this broad expanse.

For Madagascar, MCH sites were at Tritrivakely and Kavitaha [21,24]. The South American Gran Sabana contained multiple sites [11–13,54,55]) as did Karonga in Malawi, Africa [19,20]. For Australia, despite anthropological, archaeological and ecological evidence supporting palaeoanthropogenic conversion of forest to savannah [14,56], we were unable to identify a palaeocore linking contemporary grasslands at the 2000 mm MAP limit established by the framework [2], that also met the aforementioned criteria (but see electronic supplementary material, S1, for evidence of persistence of sclerophyllous systems following palaeo-human vegetation change, at MAP expected to support closed-canopy systems). In all examples, (i) forests and savannahs vacillated with Quaternary fluctuations of climate, (ii) harboured protracted charcoal spikes, with (iii) decreased woody pollen and increased grass pollen, coincident with human settlement, that was followed by (iv) permanent transformation of forest to open fire-adapted habitat, despite a return to high MAP levels.

**Figure 6.**
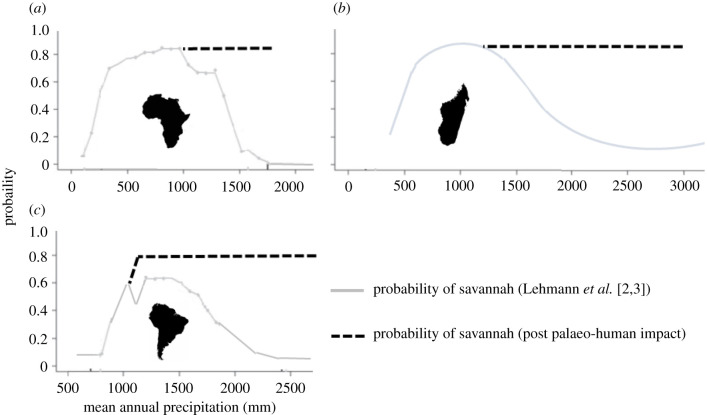
Proposed decoupling of the savannah-forest equilibrium from mean annual precipitation (MAP), at sites harbouring evidence of palaeoanthropogenic impact to habitat. (*a*) Africa, (*b*) Malagasy Central Highlands and (*c*) South America. The emergent fire-adapted grasslands do not revert to forest, even with return of high MAP [11–13,19,20,38]; adapted from Lehmann *et al.* [2,3]. (Online version in colour.)

Decoupling of MCH habitat from MAP is not an isolated example but occurs in many African and South American systems (figure 6), supporting the idea that human-induced tipping points have been crossed, preventing return of forest despite sufficient rainfall [19,20]. For the Gran Sabana, savannah exceeded 80% of cover across MAP ranges of 2000–3000 mm [11], despite the framework supporting a >90% probability of forest at MAP beyond 2500 mm for South American savannahs. For Karonga savannahs, although probability of conversion to forest for Africa exceeds 90% at MAP greater than 1750 mm, more than 80% of the area (excluding cropland) is savannah across the entire MAP range of 1000–1800 mm [38] (electronic supplementary material, S1).

## 4. Conclusion

As effects of the ‘Palaeoanthropocene’ (the period of pre-Industrial Revolution anthropogenic impacts [57]) are being discovered, they are likely to become increasingly critical to our understanding of grassland persistence at the highest MAP levels. A broad range of evidence increasingly supports impacts of the ‘palaeoanthropocene’ across continents [6,10,12,16,17,19,20,56–58]. Evidence is strong and compelling for palaeo-human disturbance dismantling the relationship between precipitation and habitat, halting the cycle of transition from open systems to closed-canopy formations [19,20]. Tipping points, once achieved, suppress forest even at the highest MAP [3,11,12,19,20]. In the context of palaeoanthropogenic habitat transformation, these data represent an important advance in understanding determinants of global savannah distribution, and acknowledge the consequence of niche construction by modern humans [20]. As more palaeocores are sampled and studied across vast systems (e.g. the Northern, Southern and Western Congolian, the Congo-Victoria basin, Trans-Fly, Cape York Peninsula, Beni, greater Guianan and Los Llanos savannahs), palaeo-human impacts may well emerge as a central consideration for persistence of contemporary savannahs. Here, we propose building upon the foundation of the current savannah framework by incorporating palaeoanthropogenic impact. It is possible that all mesic savannahs have been historically impacted by humans, potentially across vast areas. Such an approach will address the phenomenon of habitat decoupling from rainfall, provoke a new view on palaeoanthropogenic-induced savannahs and whether they are indeed ‘natural’, curtail over-estimation of MAP limits to savannah distributions and inform restoration ecology. An interesting question arises: how irrevocable is this change? As we are dealing with timescales spanning roughly 1000 to 100 000 years, an element of maintenance through ongoing anthropogenic/commensal activity may have transpired over the long-term entrenching an apparent ‘permanence’. This in turn would have important implications for conservation, as restoration should be possible, as demonstrated by expansion of tree cover following changes to farming practices in previously grass-dominated, fire-maintained agricultural systems [59].

## Data Availability

The data are provided in electronic supplementary material [60].
